# The changed endemic pattern of human adenovirus from species B to C among pediatric patients under the pressure of non-pharmaceutical interventions against COVID-19 in Beijing, China

**DOI:** 10.1186/s12985-023-01962-y

**Published:** 2023-01-09

**Authors:** Fangming Wang, Runan Zhu, Yuan Qian, Yu Sun, Dongmei Chen, Fang Wang, Yutong Zhou, Qi Guo, Liying Liu, Yanpeng Xu, Ling Cao, Dong Qu, Linqing Zhao

**Affiliations:** 1 Laboratory of Virology, Beijing Key Laboratory of Etiology of Viral Diseases in Children, Capital Institute of Pediatrics, Beijing, 100020 China; 2 Graduate School of Peking Union Medical College, Beijing, 100730 China; 3 Department of Respiratory Medicine, Affiliated Children’s Hospital, Capital Institute of Pediatrics, Beijing, 100020 China; 4 Department of Critical Care Medicine, Affiliated Children’s Hospital, Capital Institute of Pediatrics, Beijing, 100020 China

**Keywords:** Non-pharmaceutical interventions (NPIs), Human adenovirus, Endemic pattern, Types

## Abstract

**Background:**

Under the pressure of non-pharmaceutical interventions (NPIs) targeting severe acute respiratory syndrome coronavirus 2, the prevalence of human adenovirus (HAdV) was monitored before and after NPIs launched on Jan 24, 2020 in pediatric patients in Beijing, China.

**Methods:**

Respiratory samples collected from children hospitalized with acute respiratory infections from Jan 2015 to Dec 2021 were screened by direct immunofluorescence test or capillary electrophoresis-based multiplex PCR assay. The hexon, penton base, and fiber genes were amplified from HAdV positive specimens, then sequenced. For HAdV typing, phylogenetic trees were built by MEGA X. Then clinical data of HAdV positive cases were collected. All data were evaluated using SPSS Statistics 22.0 software.

**Results:**

A total of 16,097 children were enrolled and 466 (2.89%, 466/16,097) were HAdV-positive. The positive rates of HAdV varied, ranging from 4.39% (151/3,438) in 2018 to1.25% (26/2,081) in 2021, dropped from 3.19% (428/13,408) to 1.41% (38/2,689) from before to after NPIs launched (*P* < 0.001). There were 350 cases typed into nine types of species B, C, or E and 34 recorded as undetermined. Among them, HAdV-B3 (51.56%, 198/384) was the most prevalent types from 2015 to 2017, and HAdV-B7 (29.17%, 112/384) co-circulated with HAdV-B3 from 2018 to 2019. After NPIs launched, HAdV-B3 and B7 decreased sharply with HAdV-B7 undetected in 2021, while HAdV-C1 became the dominant one and the undetermined were more.

**Conclusions:**

The endemic pattern of HAdV changed in Beijing because of the NPIs launched for COVID-19. Especially, the dominant types changed from HAdV-B to HAdV-C.

**Supplementary Information:**

The online version contains supplementary material available at 10.1186/s12985-023-01962-y.

## Introduction

The outbreak of coronavirus disease 2019 (COVID-19), caused by severe acute respiratory syndrome coronavirus 2 (SARS-CoV-2), was catastrophic to the whole world. To control the pandemic of COVID-19, the strictest non-pharmaceutical interventions (NPIs), including social withdrawal, working from home, school closures, wearing masks, travel restrictions, personal hygiene improvements and border closures, had been taken in Beijing, China since Jan 24, 2020 [1]. As the COVID-19 was under control, the NPIs were downgraded from level I to level II on Apr 30, then to level III on Jul 20, 2020 in which wearing masks and social withdrawal were still required. While these NPIs effectively prevented the spread of SARS-CoV-2, they also had deeply impact on the transmission of other respiratory viruses, such as influenza viruses (Flu) [2], respiratory syncytial virus (RSV) [3], rhinovirus (Rh) [4], human metapneumovirus (HMPV) [5] and human adenovirus (HAdV) [6].

HAdV belongs to the family of *Adenoviridae* and the genus of *Mastadenovirus*, which was firstly isolated from human adenoidal tissue, and charactered as non-enveloped, non-segmented, double-stranded DNA virus with a genome length of 3.4–3.6 kb that encodes three major capsid proteins, namely, hexon, penton base and fiber [7, 8]. To date, there are 113 types of HAdVs identified and classified into seven species (A-G) [9], including 61 novel recombinant genotypes, from HAdV-D53 to HAdV-D113. It has been declared that the identification of HAdV genotypes based on partial sequence of hexon gene lead to misclassification. Therefore, the Human Adenovirus Working Group recommended that the HAdV typing should be based on the sequences of the three major capsid proteins, hexon, penton base and fiber [10].

Among the seven species of HAdVs, species B, C, and E are highly contagious and responsible for severe respiratory disease. Globally, HAdV-C1, -C2, -B3, -E4, -C5, -C6, -B7, -B14, and -B55 were the main types or genotypes in causing outbreaks [11]. In Asia, HAdV-B3 and -B7 were the predominant types associated with acute respiratory infections (ARIs) in children [11], especially among increased HAdV infections in southern China and Beijing during 2018–2019 [12, 13]. In America, HAdV-C2 was reported as most frequently detected type in HAdV surveillances [14]. HAdV-E4 was reported with sporadic circulation, but increased in many countries in recent years [15].

Under the pressure of the strictest NPIs for COVID-19, the endemic of many common pathogens for ARIs, including HAdVs, had been restrained [2–6]. However, the resurgence of respiratory viral diseases should be alerted when the measures of NPIs were relaxed [16]. To reveal the endemic pattern’s changing of HAdVs under the pressure of NPIs, the epidemiologic features of HAdVs were compared before and after NPIs launched on Jan 24, 2020.

## Methods

### Collection of clinical specimens

Clinical respiratory specimens, including throat swabs, nasopharyngeal swabs (NS), nasopharyngeal aspirates (NPAs) and bronchoalveolar lavage fluids, were collected from pediatric patients with inclusion criteria: (1) diagnosed with ARIs with signs and symptoms of fever, cough, chills, expectoration, nasal congestion, sore throat, chest pain, tachypnea, and abnormal pulmonary breath sounds[17], (2) hospitalized in respiratory department or intensive care unit, Affiliated Children’s Hospital, Capital Institute of Pediatrics during Jan 2015 to Dec 2021, (3) aged 1m ~ 14y, and exclusion criteria: (1) with more than one sample during one hospitalization, and only the first one included, (2) secondly hospitalization within 7 days.

Upon arrival at the laboratory, each clinical specimen was handled in a Class II bio-safety cabinet and processed immediately using 2.5 mL of viral transport medium (Yocon Biotechnology Co., Ltd, Beijing, China) and then centrifuged (500 × g, 10 min). Cell pellets were prepared for direct immunofluorescence (DFA) tests. Partial supernatant was used for viral nucleic acid extraction, and the remaining was stored at -80℃ for future use.

Clinical data of the enrolled patients were obtained from their electronic medical records, including age, gender, length of hospital stay, sample collection date, and clinical diagnosis.

### Respiratory pathogen screening

Respiratory specimens were subjected to DFA tests (Diagnostic Hybrids, Athens, OH, USA) for antigen testing or capillary electrophoresis-based multiplex PCR (CEMP) assay (Ningbo HEALTH Gene Technologies Ltd., Ningbo, China) for nucleic acid detection.

For DFA, cell pellets from all NS and NPAs were re-suspended and spotted onto acetone-cleaned slides. Then, individual monoclonal antibody reagents, labeled with fluorescein isothiocyanate (FITC) against HAdV, RSV, Flu A and B, and human parainfluenza virus (HPIV) 1–3 were used for virus identification under the direction of protocol manual.

For CEMP assay, total nucleic acid (DNA and RNA) was extracted firstly from 140 µL supernatant of each collected specimen using the QIAamp MinElute Virus Spin Kit (Qiagen GmbH, Germany) according to the manufacturer’s instructions. Then under the direction of manufacturer’s instructions of CEMP assay, nucleic acid was amplified in the reaction mixture containing PCR enzyme, 0.25 μM of each of the 15 pairs of primers for each targeted pathogen, dNTPs, MgCl_2_, and buffer, then subjected to capillary electrophoresis on a GeXP capillary electrophoresis system (Sciex, Concord, ON, Canada). The 15 pairs of primers detect 13 pathogens and human DNA and human RNA. The signals of the 15 labeled PCR products were measured by fluorescence. Given by the kit instructions, the positions of pathogen amplicons are as follows: Flu A 105 nt (2009H1N1 163.3 nt, H3N2 244.9 nt), HAdV 110.2/113.9 nt (represents different subtypes), human bocavirus (HBoV) 121.6 nt, Rh 129.6 nt, HPIV 181.6 nt, chlamydia (Ch) 190.5 nt, HMPV 202.8 nt, Flu B 212.7 nt, Mycoplasma pneumoniae (Mp) 217 nt, human coronavirus (HCoV) 265.1 nt, and RSV 280.3 nt. Then the left nucleic acids were preserved at − 20 °C for future use.

### HAdV typing

For specimens positive for HAdV determined by DFA or CEMP assay, the penton base, hexon, and fiber genes were amplified by polymerase chain reaction (PCR) using primer pairs, penton‐F (5′‐CTATCAGAACGACCACAGCAACTT‐3′) and penton‐R (5′‐TCCCGTGATCTGTGAGAGCRG‐3′), HVR‐F (5′-CAGGATGCTTCGGAGTACCTGAG‐3′) and HVR‐R (5′‐TTTCTGAAGTTCCACTCGTAGGTGTA‐3′), fiber‐F (5′‐CCCTC TTCCCAACTCTGGTA‐3′) and fiber‐R/CR (5′‐GGGGAGGCAAAATAACTACTCG‐3′/5′‐GAGGTGGCAGGTTGAATACTAG‐3′) [18] under the reaction conditions: 94 °C for 5 min; 94 °C for 30 s, 52 °C for 30 s, 72 °C for 1.5 min, for 45 cycles; 72 °C for 7 min. Then, PCR products were purified, and sequenced using the Sanger sequencing method by Sino Geno Max Co., Ltd. (Beijing, China). The sequences were then subjected to phylogenetic analysis to identify the HAdV types.

### Phylogenetic analysis

Lasergene’s DNA SeqMan software (version 7.1.0, DNA Star Inc. Madison, WI, USA) was used to assemble nucleotide sequences. The MAFFT software was used to align sequences of hexon, penton base and fiber genes. Phylogenetic trees were constructed by using the maximum likelihood method and 1000 bootstrap pseudo-replicates in MEGA X [19]. Reference sequences (Additional file 3: Table S1) were downloaded from GenBank.

### Statistical analysis

Jan 24, 2020 was set as the boundary of before and after NPIs launched in Beijing, China. Statistical analysis was performed using SPSS Statistics (version 22.0, IBM, NY, USA) software. In descriptive analysis, measurement data in normal distribution were presented as‾x ± s, while those in skew distribution were presented as the median and interquartile ranges (IQR). The Student’s t test, Mann–Whitney test and Chi-square (χ2) test were used for statistical analysis. Two-tailed *P*-values of < 0.05 were considered statistically significant.

## Results

### Pathogen screening

From Jan 2015 to Dec 2021, a total of 16,097 pediatric patients with ARIs were included, with the ratio of males to females 1.36:1 and median age 3.0 (1.0–5.9) years.

In DFA tests, 11,218 cases were included with 6.41% (719/11,218) positive for RSV, 2.14% (240/11,218) for HAdV, 0.65% (73/11,218) and 0.43% (48/11,218) for Flu A and Flu B, and 4.79% (537/11,218) for HPIV, respectively.

By CEMP assays, 18.45% (900/4,879) pediatric patients among 4,879 cases were determined positive for RSV, 3.56% (173/4,879) for HMPV, 4.63% (226/4,879) for HAdV, 22.13% (1,080/4,879) for Rh, 8.42% (411/4,879) for HBoV, 1.19% (58/4,879) for HCoV, 1.29% (63/4,879) and 0.67% (33/4,879) for Flu A and Flu B, and 4.25% (245/4,879) for HPIV, respectively, while 22.30% (1,088/4,879) and 1.07% (52/4,879) were positive for Mp and Ch, respectively. Except for HAdV, the other ten pathogens’ seasonal distributions based on the results of CEMP were shown in Additional file 1: Fig. S1, as well as their distributions in different age and gender groups were shown in Additional file 4: Table S2.

### The changing of HAdV endemic pattern

Among these 16,097 patients, 466 (2.89%, 466/16,097) were positive for HAdV determined by DFA or CEMP assay, including 303 males and 163 females, with a median age of 2.8 (1.3–4.4) years and varied positive rates: 1.75% (30/1,712) in 2015, 2.48% (49/1,979) in 2016, 2.73% (54/1,977) in 2017, 4.39% (151/3,438) in 2018, 3.38% (132/3,906) in 2019, 2.39% (24/1,004) in 2020, and 1.25% (26/2,081) in 2021 with the epidemic peaks in 2018 and 2019 and then significantly decreased in 2020 and 2021.

**Fig. 1:**
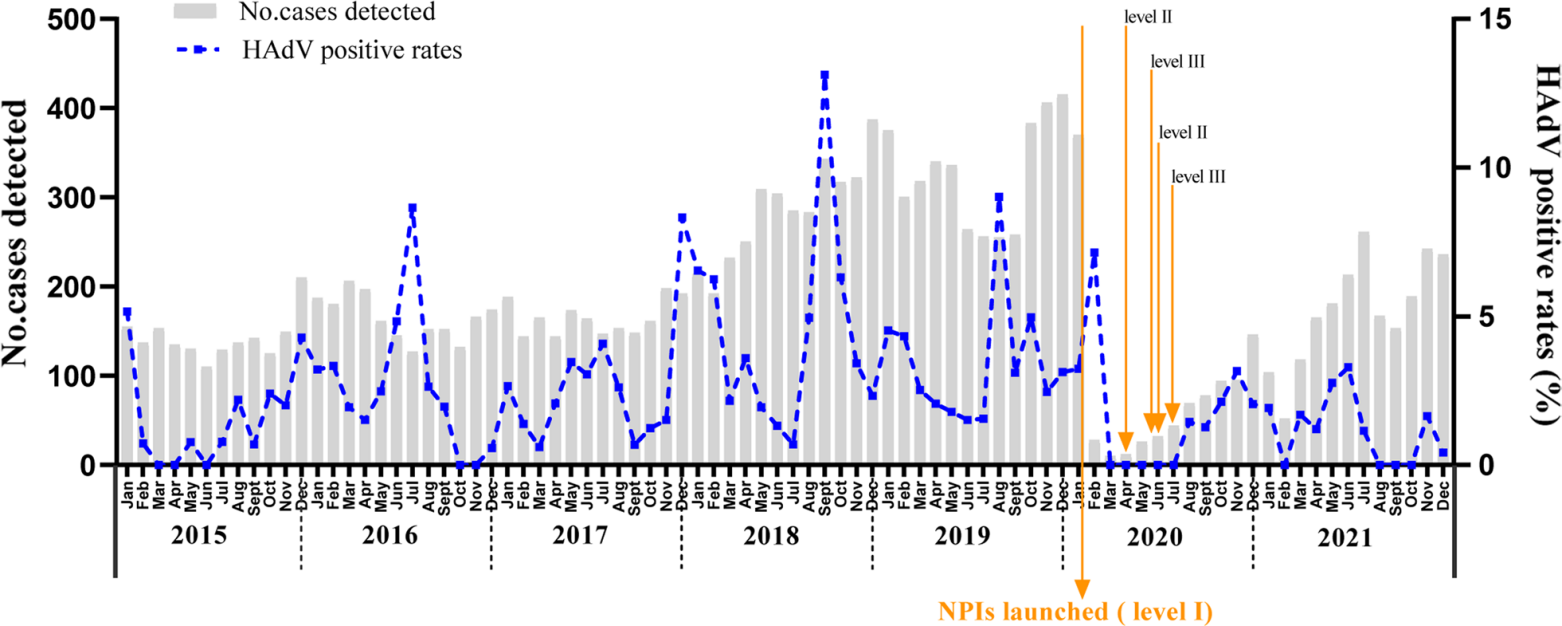
Monthly distribution of ARIs cases tested and HAdV positive rates from Jan 2015 to Dec 2021. Jan 24, 2020 was set as the boundary of before and after NPIs launched in Beijing, China

The monthly distribution of HAdV positive specimens from Jan 2015 to Dec 2021 shown in Fig. 1 revealed that the HAdV-positive rates decreased by 55.80% (*P* < 0.001) from 3.19% (428/13,408) to 1.41% (38/2,689) before and after NPIs launched. Before NPIs launched, HAdV could be detected in all months with the highest positive rates of 13.12% in Sep 2018 and 9.02% in Aug 2019. After NPIs launched, ARIs cases and HAdV-positive cases decreased sharply from Mar to Jul in 2020 with HAdV positive rates to zero, and then resurged at Aug 2020. Both the positive rates and case numbers of HAdV kept at a low level under the pressure of NPIs, even in level III of NPIs.

Among these 466 cases positive for HAdV, there were 347 cases (74.46%, 347/466) infected only by HAdV, and 119 cases also infected by other pathogens, such as Mp (21.01%, 25/119) and RSV (19.33%, 23/119), or with triple (24.37%, 29/119) or more (4.20%, 5/119) infection.

### Changes in the endemic pattern of HAdV types

**Fig. 2:**
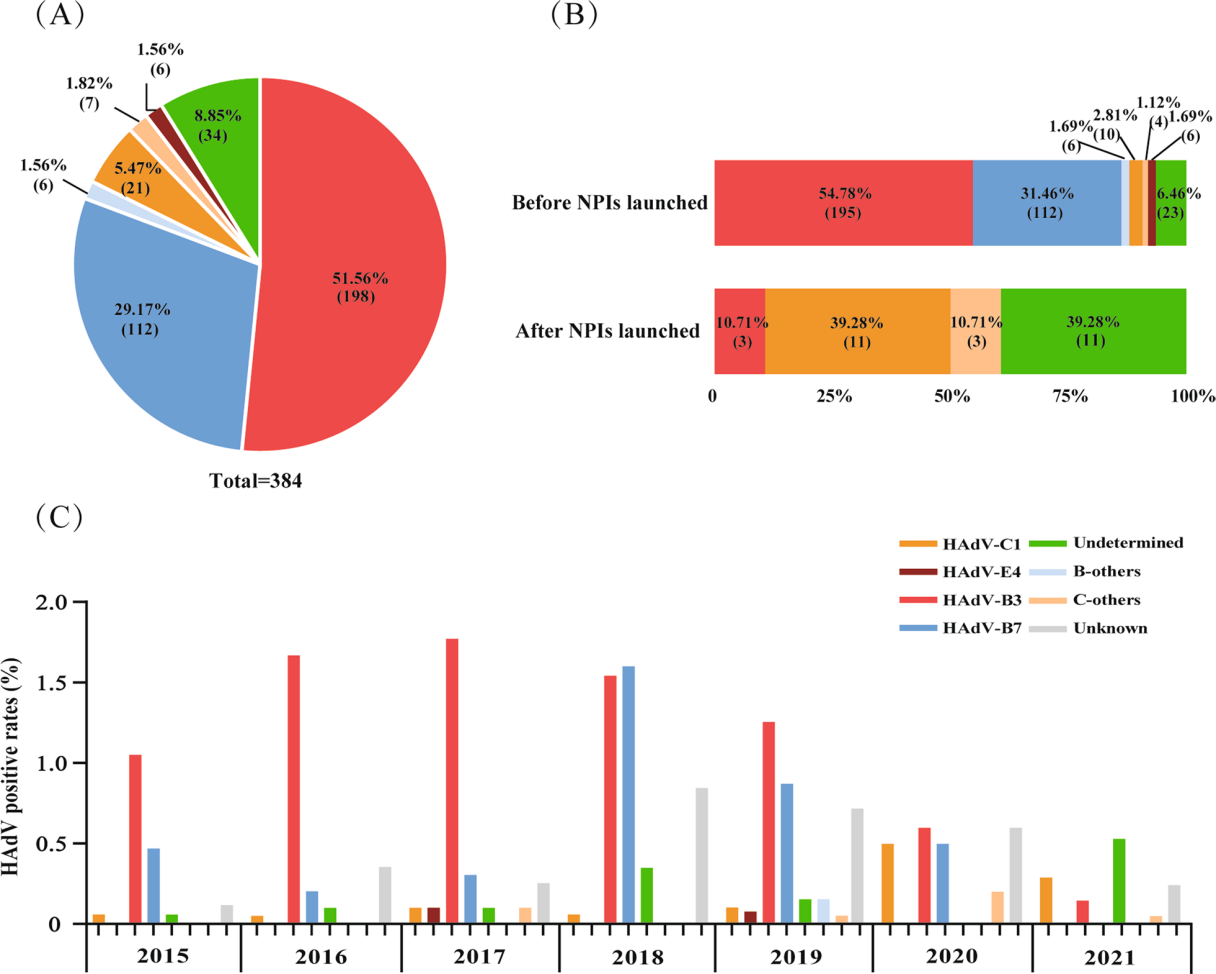
Changes in the endemic pattern of HAdV types before and after NPIs launched among HAdV-positive cases from Jan 2015 to Dec 2021, in Beijing. **A** The composition of HAdV types. **B** The composition of HAdV types among HAdV-positive cases before and after NPIs launched, respectively. **C** Positive rates of HAdV types from 2015 to 2021. “B-others” represents HAdV-B14, -B21. “C-others” represents HAdV-C2, -C5, C6. “Undetermined” represents specimens with three capsid gene sequence belonging to different types. “Unknown” represents specimens without sequences of all three capsid genes

Among specimens positive for HAdV, 75.11% (350/466) were successfully grouped into nine types and species B, C, or E by phylogenetic analysis of hexon, penton base and fiber genes (Additional file 2: Fig. S2), while 34 cases with hexon, penton base and fiber genes belonging to different HAdV types were recorded as undetermined, and the remaining 82 cases lack of enough PCR products for sequencing due to low viral load were recorded as unknown. The most prevalent types identified were HAdV-B3 (51.56%, 198/384) and HAdV-B7 (29.17%, 112/384), followed by HAdV-C1 (5.47%, 21/384) (Fig. 2A).

As shown in Fig. 2B, the predominant types of HAdV, HAdV-B3 (54.78%) and HAdV-B7 (31.46%) before NPIs launched changed to HAdV-C1 (39.28%) while more were undetermined (39.28%) after NPIs launched. As the absolute dominant one, HAdV-B3 overwhelmed HAdV-B7 from Jan 2015 to Dec 2017. Then, in 2018, HAdV-B7 (1.60%, 55/3,438) outweighed HAdV-B3 (1.54%, 53/3,438) the first time with more ARIs cases observed. For NPIs launched, the positive rate of HAdV-B3 dropped abruptly from 0.60% (6/1,004) in 2020 to 0.11% (3/2,689) in 2021, while HAdV-B7 dropped to 0.50% (5/1,004) in 2020, and then disappeared in 2021. (Fig. 2C). However, HAdV-C1 became the dominat one among HAdV positive specimens (39.28%, 11/28) after NPIs launched compared to that (5.47%, 21/384) before NPIs launched (Fig. 2B).

### Clinical characters of the HAdV-infected children

**Fig. 3:**
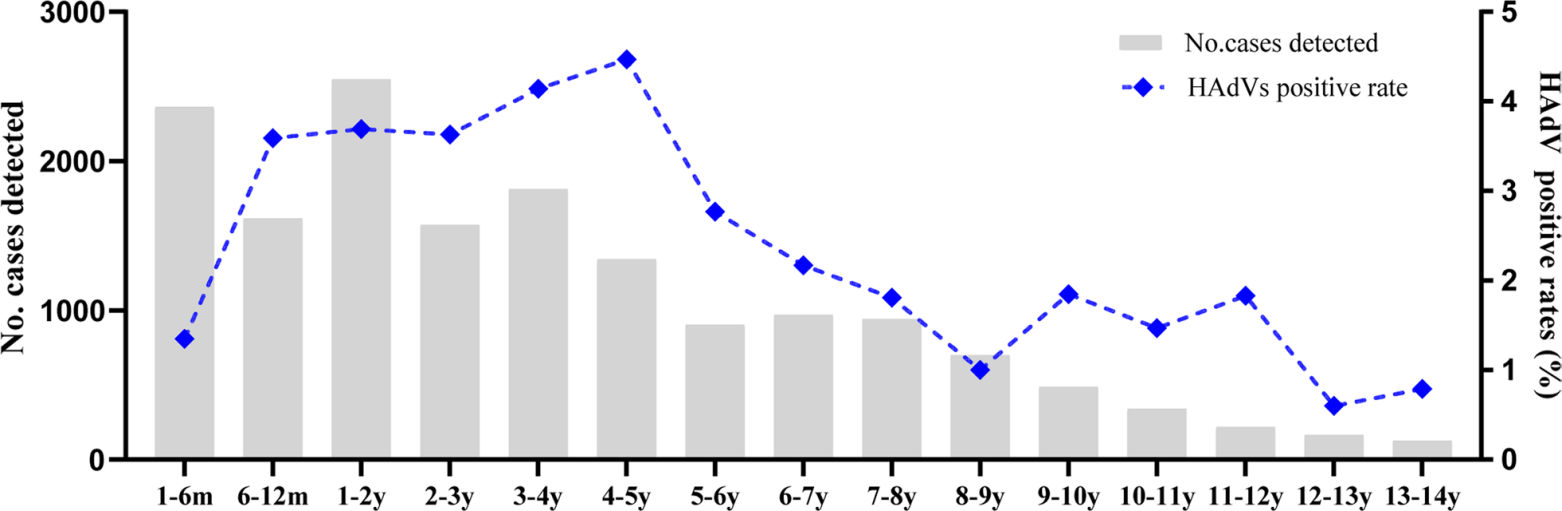
The number of ARI cases detected and positive rates of HAdV in different age groups from Jan 2015 to Dec 2021 in Beijing

Among HAdV-positive cases shown in all age groups, most of them (80.69%, 376/466) were under 5 years with positive rates 4.47% (60/1,341) in patients aged ≥ 4-5y, 4.14%(75/1,812) aged ≥ 3-4y, 3.63%(57/1,572) aged ≥ 2-3y, 3.69% (94/2,547) aged ≥ 1-2y, 3.59% (58/1,614) aged ≥ 6-12m, and 1.35% (32/2,362), the lowest one, aged ≥ 1-6m, With the increasing of age in that over 5y, the positive rates of HAdV decreased gradually, to 0.60% (1/166) in age 12-13y and 0.79% (1/126) in age 13-14y (Fig. 3).

The median length of hospital stay of HAdV positive cases after NPIs launched was 6 (4–10) d, shorter than 7 (5–13) d before NPIs launched (*P* < 0.05). No statistically significant difference was shown compared in characters of age, sex, intensive care unit admission and death among pediatric patients before and after NPIs launched (*P* > 0.05) (Table 1).

**Table 1:** Clinical characters of HAdV-infected children before and after NPIs launched

Clinical characters	Before NPIs launched (n = 428)	After NPIs launched (n = 38)	*P* value
Age, median (IQR), y	2.8 (1.3–4.5)	2.4 (1.0–4.0)	0.969^a^
Gender, Male, n (%)	275 (64.25%)	24 (63.16%)	0.893^b^
Length of stay, median (IQR), d	7 (5–13)	6 (4–10)	0.029^a^
intensive care unit admission, n (%)	69 (16.12%)	6 (15.79%)	0.957^b^
Death in the hospital, n (%)	9 (2.10%)	0	0.367^b^

*IQR* interquartile ranges

^a^Mann-Whitney test

^b^χ^2^ test

## Discussion

Under the pressure of efficient NPIs, the spread of SARS-CoV-2 has been controlled effectively [20]. HAdV is similar with SARS-CoV-2 in the main route of transmission through respiratory tract. Therefore, it was reported that the prevalence of HAdV during the COVID-19 pandemic has changed significantly because of NPIs launched [6]. In the study, the prevalence of HAdV within seven years in pediatric patients in Beijing, China before and after NPIs launched was continuously monitored.

Although there is no obvious seasonal distribution of HAdV reported, the endemic season of HAdV often occurs in winter or early spring in north China [21]. The results in the study during Jan 2015 to Dec 2021 indicated that the highest positive rates of HAdV occurred in 2018 (4.39%, 151/3,438) and 2019 (3.38%, 132/3,906), especially in Sep 2018 (13.12%) and Aug 2019 (9.02%), while more cases of ARIs (3,438 in 2018. and 3,906 in 2019). Then the positive rates of HAdV dropped to about zero in the next five months after Feb 2020, NPIs launched (Fig. 1). When COVID-19 was under control in Beijing, China, from Jul 2020 to 2021, NPIs were adjusted to level III, in which epidemic prevention measures were taken regularly, and wearing masks in public was mandatory. However, the incidence of HAdV did not increase as expected, but kept in declining in 2021, to the lowest value in recent seven years.

Accumulated data revealed that under the NPIs targeting SARS-CoV-2, the epidemic trends of respiratory viruses had changed, such as the long-term dormancy period of Flu [2], the delay of RSV epidemic season [3] and the surge of Rh [4]. The survey of children in Guangzhou showed that the HAdV positive cases decreased significantly from 2020 to 2021 due to the NPIs against SARS-CoV-2 [22]. Low levels of HAdV prevalence also were shown in another survey in Beijing among children and adults. In addition, it was reported that the prevalence of HAdV kept in low level through the whole 2020 in US, till to early May 2021 [23]. Brauner et al. [24] analyzed the impact of COVID-19 in 41 countries, and confirmed that NPIs were effective in alleviating and inhibiting COVID-19. A recent survey conducted in Hong Kong showed that 85% of respondents were used to avoid crowded places, and 99% were used to wearing masks when leaving home [25]. These data confirmed the powerful roles of NPIs in controlling the transmission of respiratory viruses.

Before NPIs launched, HAdV-B3 and HAdV-B7 were the predominant types in Beijing. From 2015 to 2017, HAdV-B3 was the dominant one, and from 2018 to 2019, HAdV-B3/B7 circulated together. The high prevalence of HAdV between 2018 and 2019 was the result of the raising of HAdV-B7. After NPIs launched in Beijing on Jan 24, 2020, the positive rates of HAdV-B3 and HAdV-B7 dropped sharply, with that of HAdV-B7 finally dropped to zero in 2021 (Fig. 2). However, with the declining of HAdV-B, HAdV-C1 became dominant type, which revealed an endemic pattern changing of HAdV from species B to C. Another prospective surveillance study among children and adults in Beijing with HAdV associated ARIs has revealed the disappearance of species B during the COVID-19 pandemic [26]. HAdV-B3 and B7 usually were the most common types of ARIs in China, and HAdV-B7 was associated with more severe disease than that of HAdV-B3 and other HAdV types [27]. Therefore, it was necessary to keep an alert on the reemergence of HAdV-B3/B7 in the future. HAdV-C, an uncommon type in hospitalized children [28] and usually causing milder clinical symptoms [29], could cause an asymptomatic persistent infection after initial infection [30], which may explain the high proportion of HAdV-C among HAdV positive cases after NPIs launched.

In addition, there were 34 cases, including 23 and 11 collected before and after NPIs launched, respectively, with sequences of hexon, penton base and fiber genes belonging to different HAdV types, which still cannot be undetermined as recombinants or co-infections of different types in the study. Those collected after NPIs launched accounted for a higher proportion (39.28%) of HAdV positive cases. More data should be accumulated to reveal the meaning of the high proportion of recombinants or co-infections.

Children under 5 years old were the main population for HAdV infections in inpatients in Beijing. The length of hospital stay of pediatric patients was shorter (*P* < 0.05), and no death case was reported after NPIs launched. Nijman et. al. [31] showed that the parental health-seeking behaviors have changed during the COVID-19 pandemic. In China, the timely diagnosis and sufficient medical resources might contribute to the decreased RSV-associated disease severity during COVID-19 [32].

All these results had important public health implications. Under the pressure of NPIs, the positive rates of HAdVs dropped obviously and kept in low level in 2021. It’s the first report that the endemic pattern of HAdVs changed from HAdV-B to HAdV-C in children in Beijing. More survey should be done to monitor the endemic pattern of HAdV in order to keep on alert for the reemergence of HAdV-B and prevent large outbreaks in the future. However, there were several limitations in the research. First, extended studies are needed to confirm the present findings harvested from a single-center study. Secondly, there were 32 cases undetermined as recombinants or co-infections of different HAdV types. Thirdly, there were 82 cases cannot be sequenced for low viral load.

## Conclusions

The endemic pattern of HAdV was changed in Beijing because of the NPIs launched for SARS-CoV-2, including the declining of both ARIs and HAdV positive cases, while the dominant types changed from HAdV-B to HAdV-C, the higher proportion of cases undetermined, and milder clinical outcome.

## Supplementary Information

**Additional file 1: Fig. S1**. Monthly distributions of ten pathogens from Jan 2017 to Dec 2021 based on the results of CEMP assays. Jan 24, 2020 was set as the boundary of before and after NPIs launched in Beijing, China. (**A**) Respiratory syncytial virus(RSV). (**B**) Influenza A virus (Flu A). (**C**) Influenza B virus (Flu B). (**D**) Human parainfluenza virus (HPIV). (**E**) Human metapneumovirus (HMPV). (**F**) Rhinovirus (Rh). (**G**) Human bocavirus (HBoV). (**H**) Human coronavirus (HCoV). (**I**) Mycoplasma pneumoniae (Mp). (**J**) Chlamydia (Ch).

**Additional file 2: Fig. S2**. The phylogenetic trees of hexon (**A**), penton base (**B**), and fiber genes (**C**), respectively, of the 350 identified HAdV-positive clinical specimens, generated on the General Time Reversible (GTR) model by MEGA × using the maximum parsimony method with 1,000 boot-strap replicates. Reference sequences were downloaded from GenBank and labeled with blue.

**Additional file 3: Table S1**. List of reference HAdV strains used in the manuscript’s hexon, penton base and fiber genes phylogenetic comparisons.

**Additional file 4: Table S2**. Numbers of children infected with ten pathogens in different age and gender groups.

## Abbreviations

COVID-19: Coronavirus disease 2019
SARS-CoV-2: Severe acute respiratory syndrome coronavirus 2
NPIs: Non-pharmaceutical interventions
ARIs: Acute respiratory infections
HAdV: Human adenovirus
Flu: Influenza viruses
RSV: Respiratory syncytial virus
Rh: Rhinovirus
HMPV: Human metapneumovirus
HPIV: Human parainfluenza virus
HBoV: Human bocavirus
HCoV: Human coronavirus
Ch: Chlamydia
Mp: Mycoplasma pneumoniae
NS: Nasopharyngeal swabs
NPAs: Nasopharyngeal aspirates
DFA: Direct immunofluorescence
CEMP: Capillary electrophoresis-based multiplex PCR
IQR: Interquartile ranges
