# Supplementary figures and images for: The changed endemic pattern of human adenovirus from species B to C among pediatric patients under the pressure of non-pharmaceutical interventions against COVID-19 in Beijing, China
Source: Virol J. 2023 Jan 9;20:4. doi: 10.1186/s12985-023-01962-y (PMC9828375; doi:10.1186/s12985-023-01962-y)

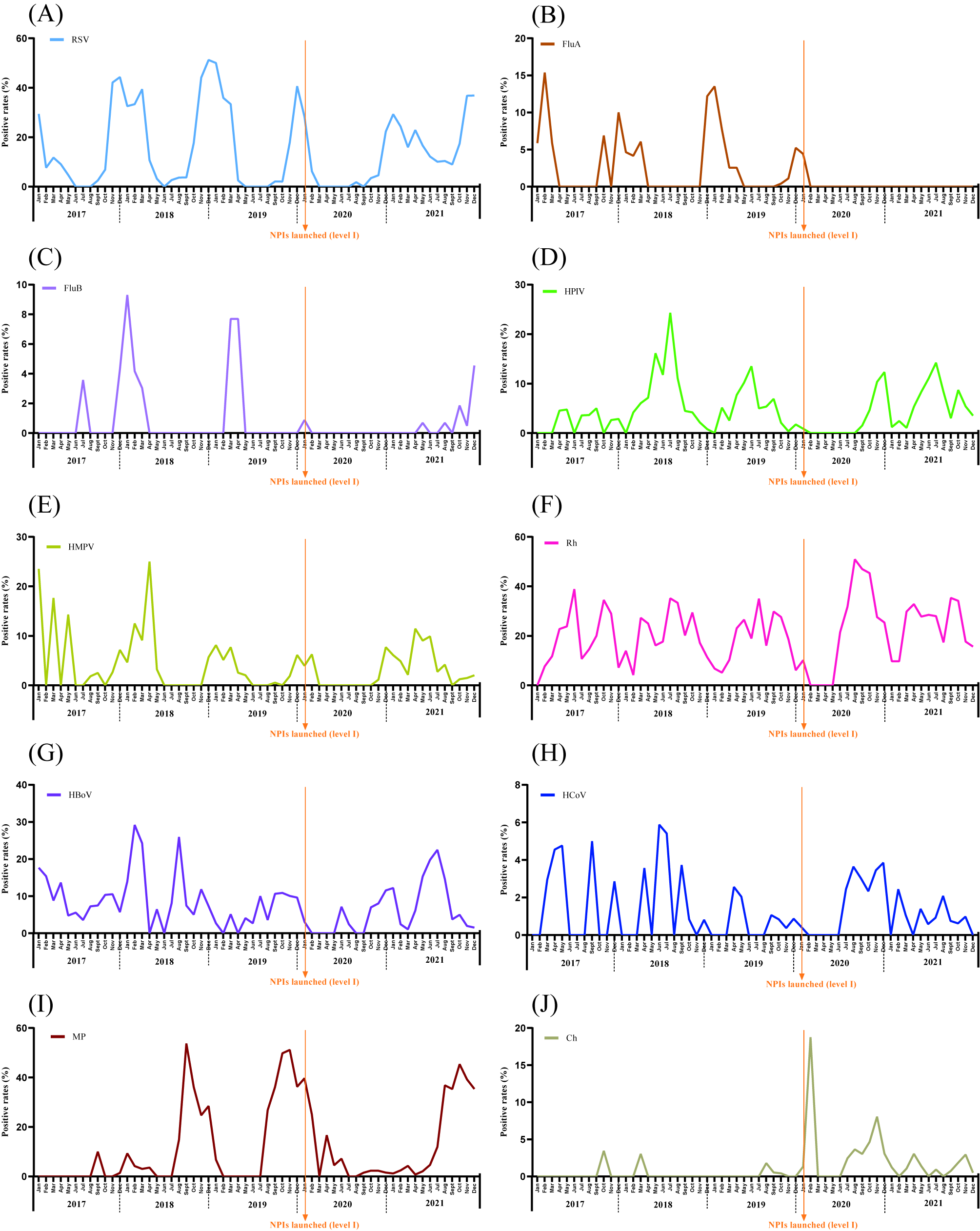

Supplement: Supplementary file 1 — Additional file 1: Fig. S1. Monthly distributions of ten pathogens from Jan 2017 to Dec 2021 based on the results of CEMP assays. Jan 24, 2020 was set as the boundary of before and after NPIs launched in Beijing, China. (A) Respiratory syncytial virus(RSV). (B) Influenza A virus (Flu A). (C) Influenza B virus (Flu B). (D) Human parainfluenza virus (HPIV). (E) Human metapneumovirus (HMPV). (F) Rhinovirus (Rh). (G) Human bocavirus (HBoV). (H) Human coronavirus (HCoV). (I) Mycoplasma pneumoniae (Mp). (J) Chlamydia (Ch). [file 12985_2023_1962_MOESM1_ESM.tif]

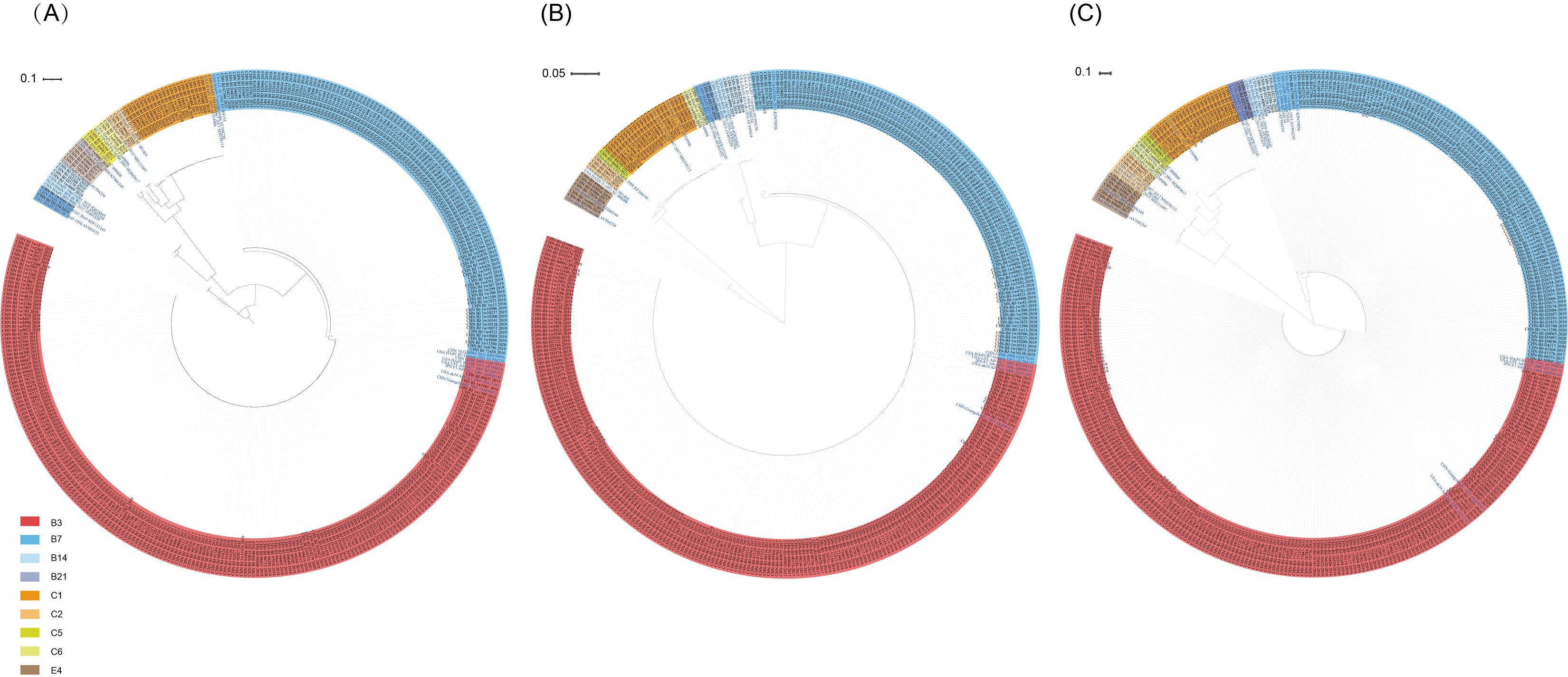

Supplement: Supplementary file 2 — Additional file 2: Fig. S2. The phylogenetic trees of hexon (A), penton base (B), and fiber genes (C), respectively, of the 350 identified HAdV-positive clinical specimens, generated on the General Time Reversible (GTR) model by MEGA × using the maximum parsimony method with 1,000 boot-strap replicates. Reference sequences were downloaded from GenBank and labeled with blue. [file 12985_2023_1962_MOESM2_ESM.tif]
