# Supplementary material for: The changed endemic pattern of human adenovirus from species B to C among pediatric patients under the pressure of non-pharmaceutical interventions against COVID-19 in Beijing, China
Source: Virol J. 2023 Jan 9;20:4. doi: 10.1186/s12985-023-01962-y (PMC9828375; doi:10.1186/s12985-023-01962-y)
Supplement: Supplementary file 3 — Additional file 3: Table S1. List of reference HAdV strains used in the manuscript’s hexon, penton base and fiber genes phylogenetic comparisons. [file 12985_2023_1962_MOESM3_ESM.docx]

| **GenBank ID** | **Organism** | **Country of origin** | **Collection date** | **Strain Name** |
| --- | --- | --- | --- | --- |
| AY599834 | Human adenovirus B3 | USA | 1953 | Strain GB |
| JX423382 | Human adenovirus B3 | USA | 2008 | ak34_AdV3a2 |
| DQ099432 | Human adenovirus B3 | CHN | 2005 | Guangzhou01 |
| AB900148 | Human adenovirus B3 | JPN | 1988 | F1_Adv3a52 |
| KF268131 | Human adenovirus B3 | USA | 2007 | UFL_Adv3a17 |
| AY594255 | Human adenovirus B7 | USA | 1953 | Gomen |
| KP670856 | Human adenovirus B7d | CHN | 2011 | GZ6965 |
| AY594256 | Human adenovirus B7 | USA | - | Vaccine strain |
| GQ478341 | Human adenovirus B7 | CHN | 2008 | GZ08 |
| KF268134 | Human adenovirus B7 | USA | 1988 | CL_43 |
| AY594255 | Human adenovirus B7 | USA | 1953 | Gomen |
| AY803294 | Human adenovirus B14p | NLD | 1955 | de_Wit |
| JQ824845 | Human adenovirus B14p1 | CHN | 2010 | CHN/GZ01 |
| JX491639 | Human adenovirus B55 | CHN | 2011 | BJ01 |
| MW151243 | Human adenovirus B21 | CHN | 2019 | GZ09107 |
| AY601633 | Human adenovirus B21 | SAU | 1956 | AV-1645 |
| AF534906 | Human adenovirus C1 | USA | 1953 | HAdV-1 |
| NC_001405 | Human adenovirus C2 | USA | 1953 | HAdV-2 |
| AC_000008 | Human adenovirus C5 | USA | 1953 | HAdV-5 |
| KF268199 | Human adenovirus C5 | USA | 2008 | UFL_Adv5 |
| FJ349096 | Human adenovirus C6 | USA | 1953 | HAdV-6 |
| HQ003817 | Human adenovirus C57 | RUS | 2001 | 16700 |
| MH121097 | Human adenovirus C89 | DEU | 2015 | 29C2 |
| MH558113 | Human adenovirus C104 | CHN | 2017 | GD2467 |
| KF006344 | Human adenovirus E4 | CHN | 2008 | GZ01 |
| AY594254 | Human adenovirus E4 | USA | - | Vaccine strain |

**Supplementary Table 1.** List of reference human adenovirus (HAdV) strains used in the manuscript’s hexon, penton base and fiber genes phylogenetic comparisons.
