# Supplementary material for: The changed endemic pattern of human adenovirus from species B to C among pediatric patients under the pressure of non-pharmaceutical interventions against COVID-19 in Beijing, China
Source: Virol J. 2023 Jan 9;20:4. doi: 10.1186/s12985-023-01962-y (PMC9828375; doi:10.1186/s12985-023-01962-y)
Supplement: Supplementary file 4 — Additional file 4: Table S2. Numbers of children infected with ten pathogens in different age and gender groups. [file 12985_2023_1962_MOESM4_ESM.docx]

Table S2. Numbers of children infected with ten pathogens in different age and gender groups

| Pathogens | RSV | hMPV | Rh | HBoV | HCoV | FluA | FluB | PIV | MP | Ch |
| --- | --- | --- | --- | --- | --- | --- | --- | --- | --- | --- |
| Total (n.) | 900 | 173 | 1080 | 411 | 58 | 63 | 33 | 245 | 1088 | 52 |
| Age groups |  |  |  |  |  |  |  |  |  |  |
| 1m-1y (n.) | 515 | 54 | 347 | 94 | 26 | 23 | 10 | 102 | 33 | 36 |
| 1-3y (n.) | 198 | 45 | 313 | 203 | 21 | 19 | 9 | 83 | 143 | 4 |
| 3-6y (n.) | 155 | 60 | 301 | 102 | 7 | 16 | 12 | 48 | 322 | 6 |
| ＞6y (n.) | 32 | 14 | 119 | 12 | 4 | 5 | 2 | 12 | 590 | 6 |
| Gender |  |  |  |  |  |  |  |  |  |  |
| Male (n.) | 537 | 99 | 694 | 253 | 38 | 38 | 18 | 156 | 579 | 30 |
| Female (n.) | 363 | 74 | 386 | 158 | 20 | 25 | 15 | 89 | 509 | 22 |
